# Supplementary material for: T-cell deficiency and hyperinflammatory monocyte responses associate with Mycobacterium avium complex lung disease
Source: Front Immunol. 2022 Oct 3;13:1016038. doi: 10.3389/fimmu.2022.1016038 (PMC9574438; doi:10.3389/fimmu.2022.1016038)
Supplement: Supplementary file 1 [file DataSheet_1.pdf]

Supplemental material

T-cell deficiency and hyperinflammatory monocyte responses associate with MAC lung disease

Cecilia S. Lindestam Arlehamn[1,*], Basilin Benson[2], Rebecca Kuan[1], Kimberley A. Dill-McFarland[2], Glenna J. Peterson[2], Sinu Paul[1], Felicia K. Nguyen[2], Robert H. Gilman[3,4], Mayuko Saito[5], Randy Taplitz[6], Matthew Arentz [7,8], Christopher H. Goss[2], Moira L. Aitken[2], David J. Horne[2,7], Javeed A. Shah[2,9], Alessandro Sette[1,10], Thomas R. Hawn[2]

[1] Center for Infectious Disease and Vaccine Research, La Jolla Institute for Immunology, La Jolla, CA, USA

[2] Department of Medicine, University of Washington, Seattle, WA, USA

[3] Johns Hopkins School of Public Health, Baltimore, MD, USA

[4] Universidad Peruana Cayetano Heredia, Lima, Peru

[5] Department of Virology, Tohoku University Graduate School of Medicine, Sendai, Japan

[6] Department of Medicine, City of Hope National Medical Center, Duarte, CA, USA

[7] Department of Global Health, University of Washington, Seattle. WA, USA

[8] FIND, the global alliance for diagnostics, Geneva, Switzerland

[9] VA Puget Sound Healthcare System, Seattle, WA, USA

[10] Department of Medicine, University of California San Diego, La Jolla, CA, USA

Supplemental Figures and Legends

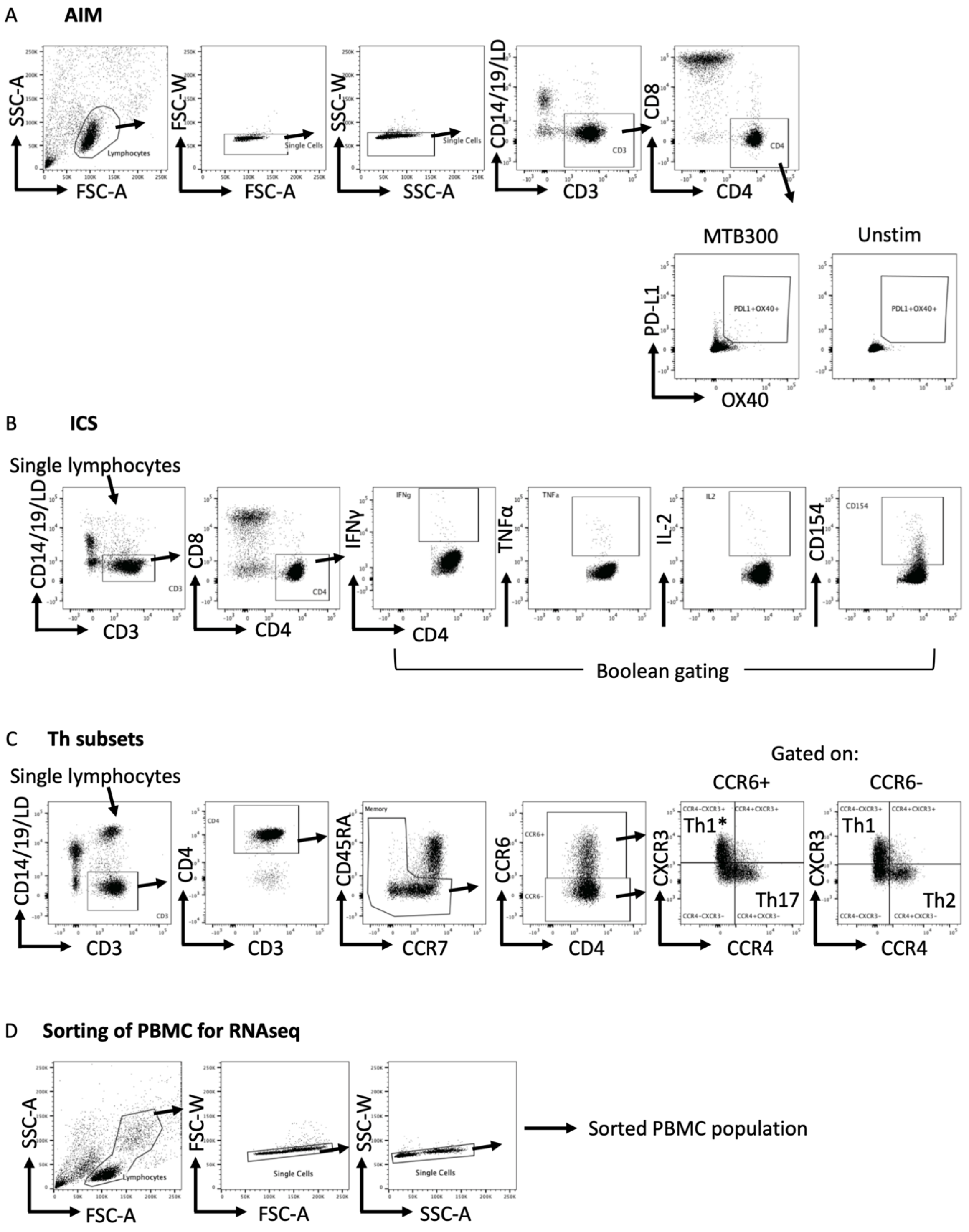

Figure S1. Gating strategy for flow cytometry experiments. A) AIM assay, B) ICS assay, C) Th subset determination, D) Sorting strategy. A-C) Live/Dead stain (LD), B,C) The single lympohocyte population was gated based on forward and side-scatter parameters.

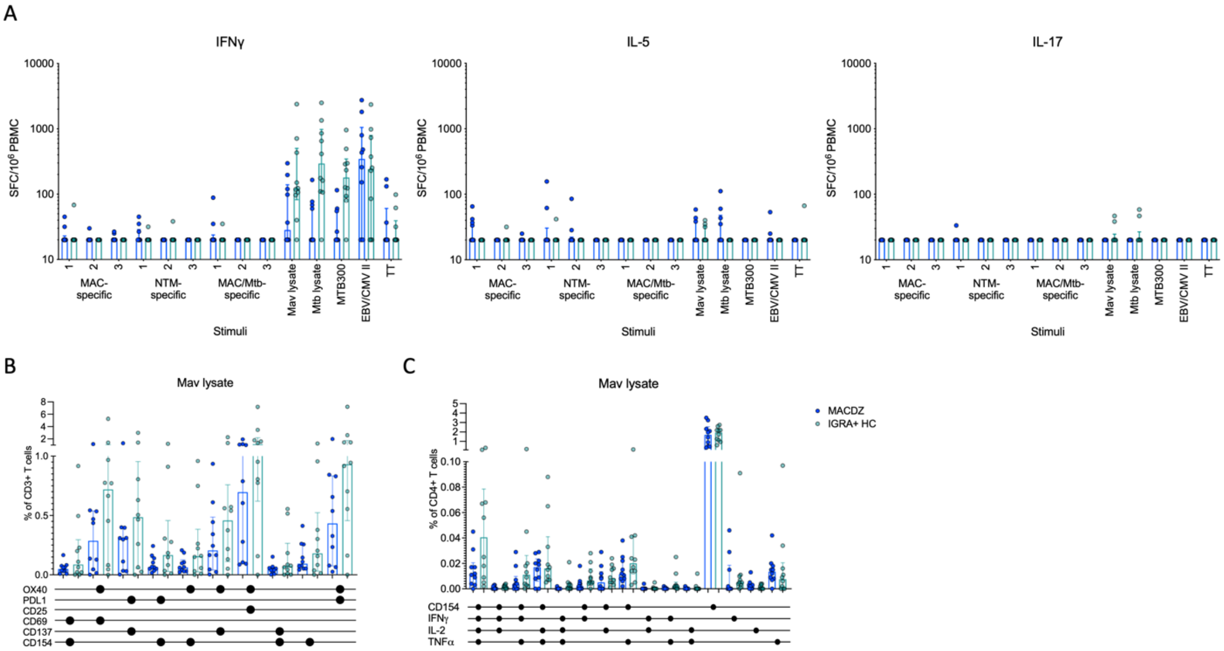

Figure S2. Individuals with MAC disease have infrequent Mav- or mycobacteria-specific responses. A) IFNγ, IL-5 or IL-17-specific magnitude of response against peptide pools, Mav and Mtb lysates as SFC per 10^6 cultured PBMC as determined by Fluorospot. Each point represents one participant (MACDZ, n=10 in blue; IGRA+HC, n=10 in teal), median ± interquartile range is shown. B) Frequency of indicated combinations of activation markers in CD4 T-cells in response to Mav lysate shown as % of CD3+ T-cells. Each point represents one participant (MACDZ, n=10; IGRA+HC, n=10), median ± interquartile range is shown. Two-tailed Mann-Whitney test is >0.05 for all stimuli comparing MACDZ vs. IGRA+HC. CD4 T-cells were gated as CD3+CD4+CD8-CD19-CD14- in the live singlet gate of PBMC. C) Frequency of indicated combinations of CD154, IFNγ, IL-2 and TNFα in CD4 T-cells in response to Mav lysate. Each point represents one participant (MACDZ, n=10; IGRA+HC, n=10), median ± interquartile range is shown. Combinations of cytokines were determined by Boolean gating following the gating strategy in Figure S1.

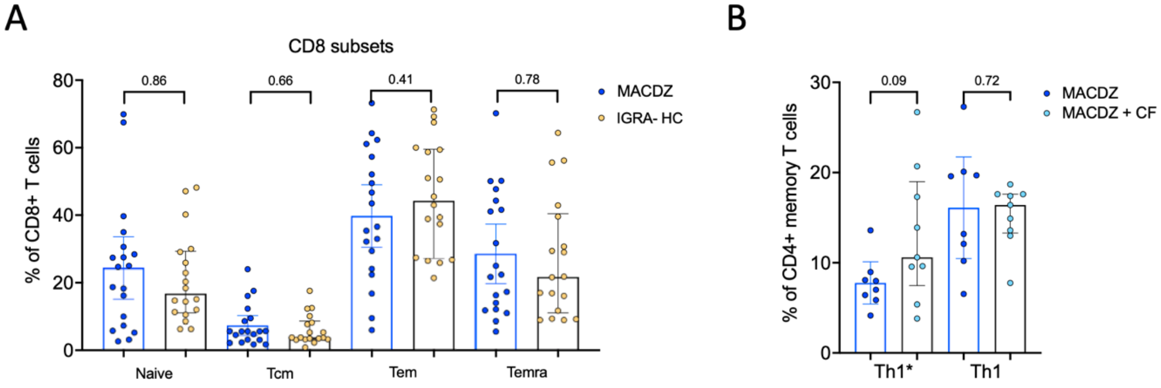

Figure S3. Individuals with MAC disease have similar frequencies of CD8 memory populations and no difference if they have cystic fibrosis of specific cell subsets. A) Frequency of CD8 memory populations based on CD45RA and CCR7 expression divided in naïve, effector memory (Tem), central memory (Tcm) and Temra populations. Each point represents one participant (MACDZ, n=19 in blue, IGRA-HC, n=18 in yellow), median ± interquartile range is shown. Two-tailed Mann-Whitney test. B) Frequency of specific Th subsets (Th1* and Th1) in MACDZ individuals with (MACDZ+CF, n=9 in light blue) or without cystic fibrosis (MACDZ, n=8, in dark blue). Each point represents one participant, median ± interquartile range is shown. Two-tailed Mann-Whitney test.

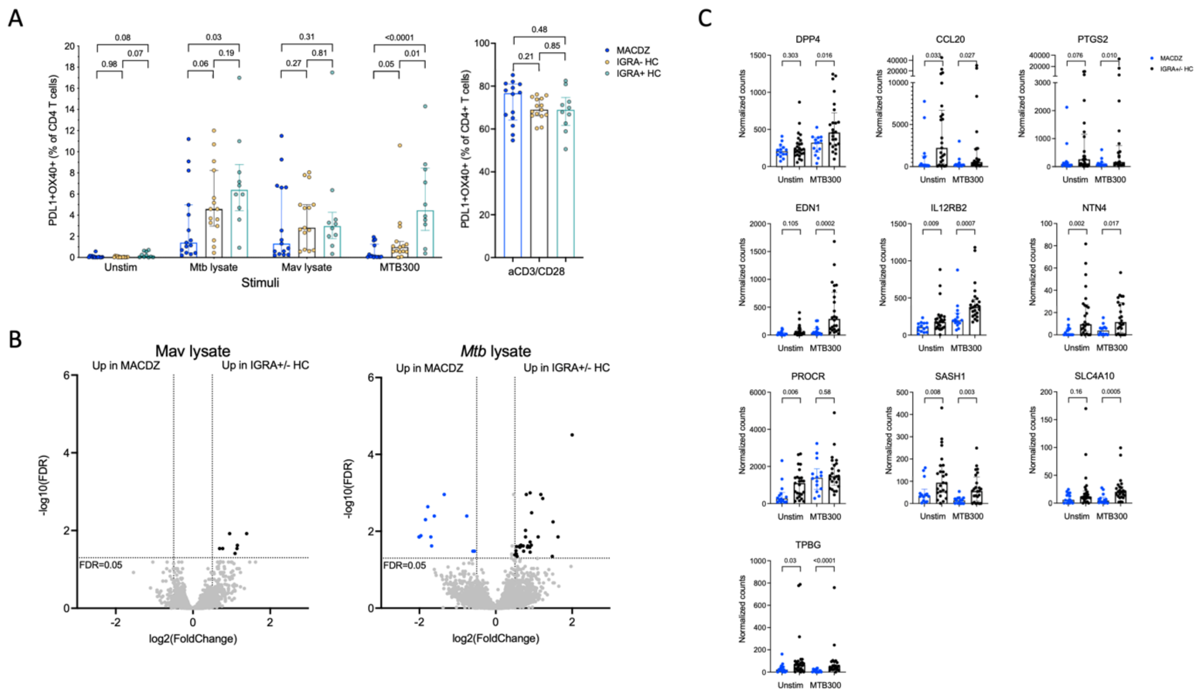

Figure S4. Activation induced marker upregulation in RNAseq cohorts, and differentially expressed genes in response to Mav and Mtb lysates. A) Frequency of PDL1+OX40+ CD4 T-cells in response to MTB300, Mav and Mtb lysates, and anti-CD3/CD28 stimulation. Each point represents one participant (MACDZ, n=15; IGRA-HC, n=10, and IGRA+HC, n=10), median ± interquartile range is shown. Two-tailed Mann-Whitney test. CD4 T-cells were gated as CD3+CD4+CD8-CD19-CD14- in the live singlet gate of PBMC. B) Volcano plots showing differentially expressed genes in Mav and Mtb lysate stimulated samples comparing individuals with MAC disease (upregulated genes shown in blue) to IGRA+/-HC individuals (upregulated genes shown in black). Adjusted p-value <0.05 and log2 fold change >0.5 or <-0.5, Benjamini Hochberg corrected DESeq2 Wald test. C) Th1* signature gene expression in the unstimulated or MTB300 stimulated MACDZ and IGRA+/-HC samples. Each point represents one participant, median ± interquartile range is shown. Two-tailed Mann-Whitney test.

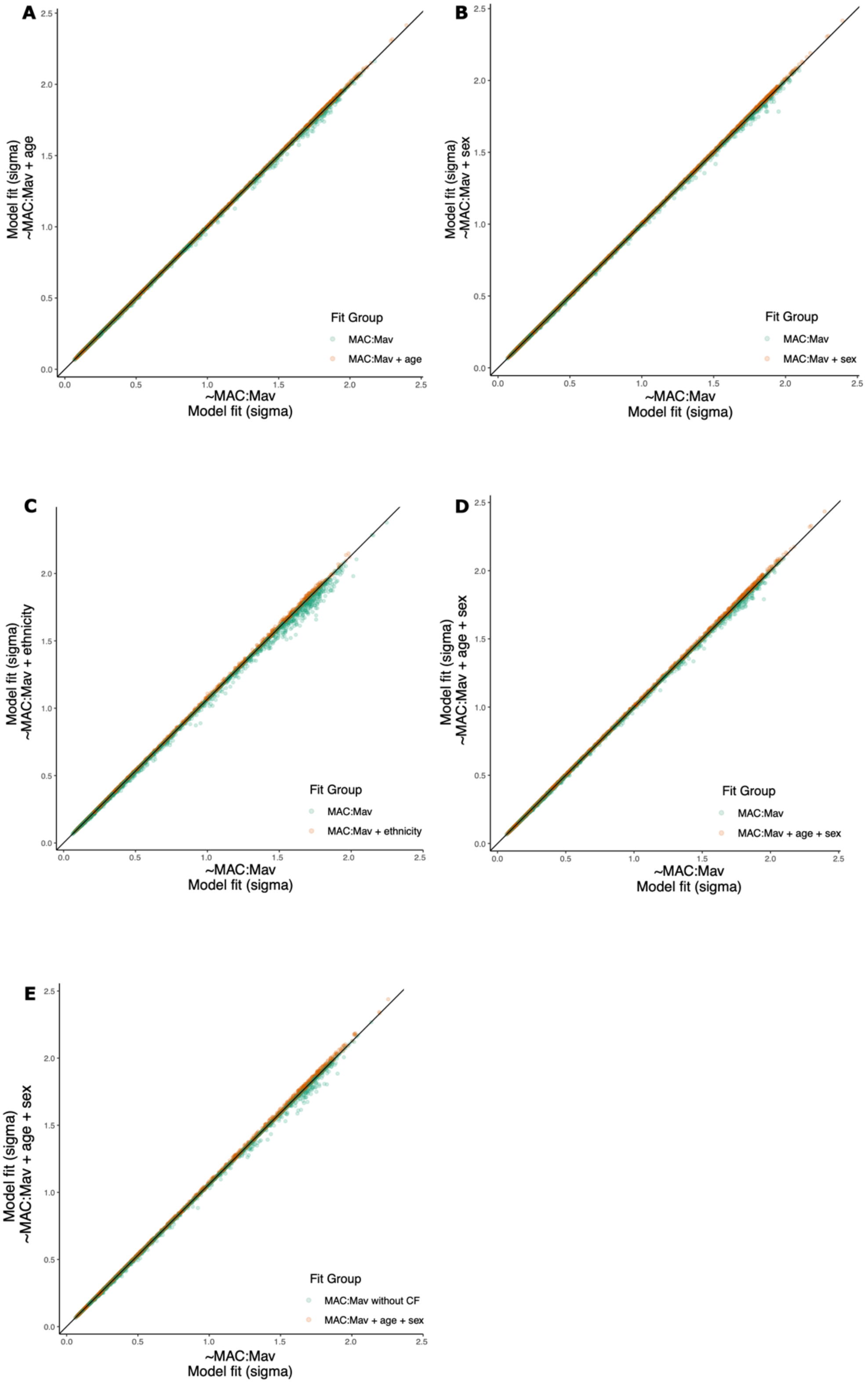
A
Model fit (sigma) ~MAC:Mav + age
~MAC:Mav Model fit (sigma)
Fit Group
MAC:Mav
MAC:Mav + age
B
Model fit (sigma) ~MAC:Mav + sex
~MAC:Mav Model fit (sigma)
Fit Group
MAC:Mav
MAC:Mav + sex
C
Model fit (sigma) ~MAC:Mav + ethnicity
~MAC:Mav Model fit (sigma)
Fit Group
MAC:Mav
MAC:Mav + ethnicity
D
Model fit (sigma) ~MAC:Mav + age + sex
~MAC:Mav Model fit (sigma)
Fit Group
MAC:Mav
MAC:Mav + age + sex
E
Model fit (sigma) ~MAC:Mav + age + sex
~MAC:Mav Model fit (sigma)
Fit Group
MAC:Mav without CF
MAC:Mav + age + sex

Figure S5. Sigma plots from linear model comparing RNASeq transcriptional profiles in MACDZ and HC subjects. Expression profiles were compared between MACDZ and HC subjects with and without Mav infection using a linear model that incorporated an interaction term in addition to the main effects: Expression ~ phenotype + stimulation + phenotype:stimulation and Expression ~ MACDZ + Mav + MACDZ:Mav +/- covariates with patient included as random effects and age, sex, and ethnicity included as covariates using R packages lme4. (A-C) Sigma plots indicate that inclusion of age **(A)**, sex **(B)**, or ethnicity **(C)** as covariates in the model did not improve the fit (median sigma changes 0.0001 for age, 0.00007 for sex, and 0.000003 for ethnicity. To assess the effect of CF, we examined sigma plots of a model including age and sex as covariates with **(D)** and without **(E)** CF samples. The model fit did not improve with including age, sex, or ethnicity as covariates or with the exclusion of CF samples.

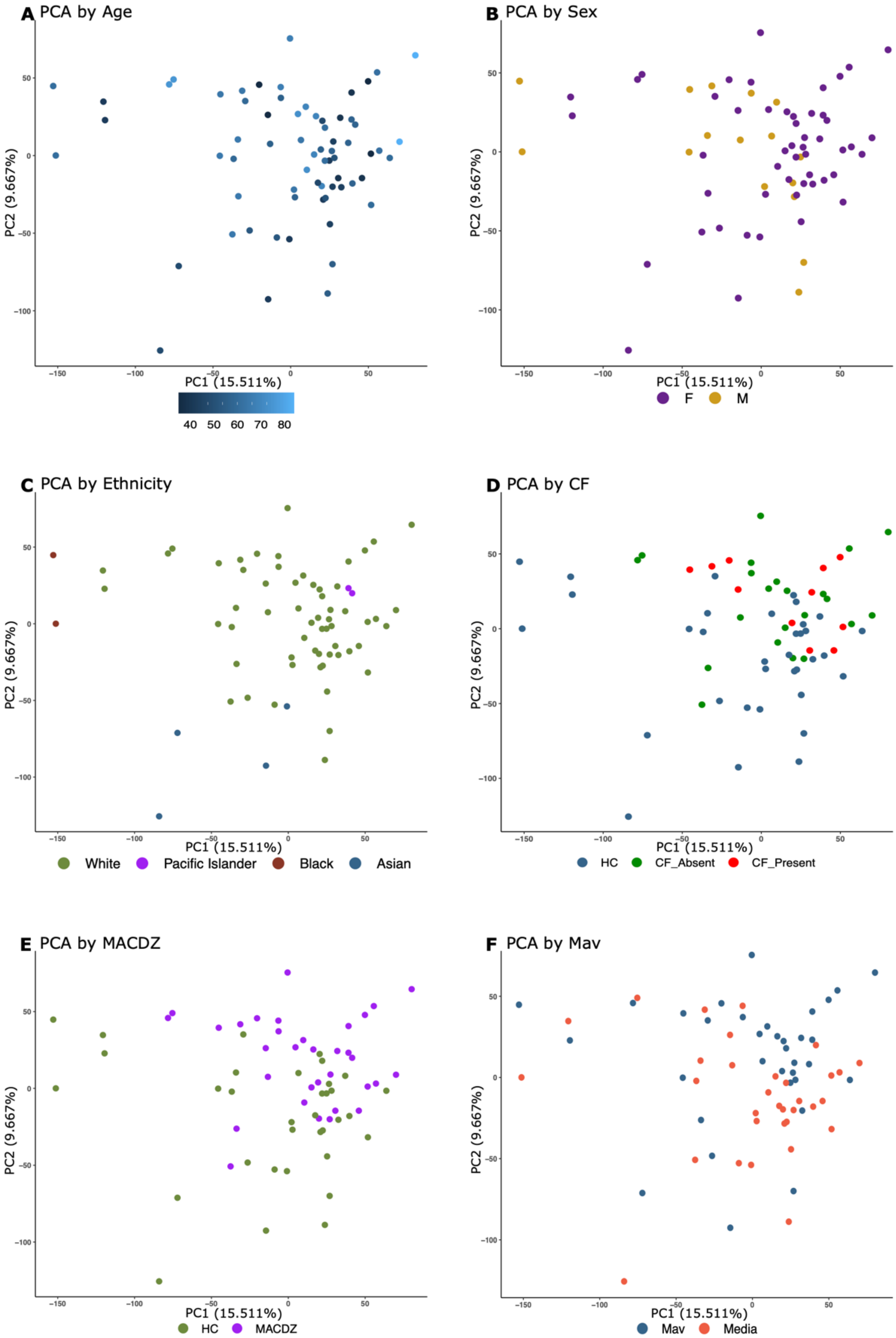
A PCA by Age
B PCA by Sex
C PCA by Ethnicity
D PCA by CF
E PCA by MACDZ
F PCA by Mav
PC1 (15.511%)
PC2 (9.667%)
40 50 60 70 80
F M
White Pacific Islander Black Asian
HC CF_Absent CF_Present
HC MACDZ
Mav Media

Figure S6. Principal component analysis (PCA) of RNASeq profiles of monocyte response to *Mycobacterium avium* infection in MACDZ versus HC subjects.

Principal component analysis (PCA) plots of monocyte RNASeq transcriptional profiles from MACDZ subjects (N=17) or HC (N=17) with media only condition or after infection with *M. avium* (MOI=5) for 6 hours. PCA colored by age **(A)**, sex **(B)**, ethnicity **(C)**, cystic fibrosis **(D),** MACDZ vs HC **(E)**, or media vs M. avium condition **(F)**.

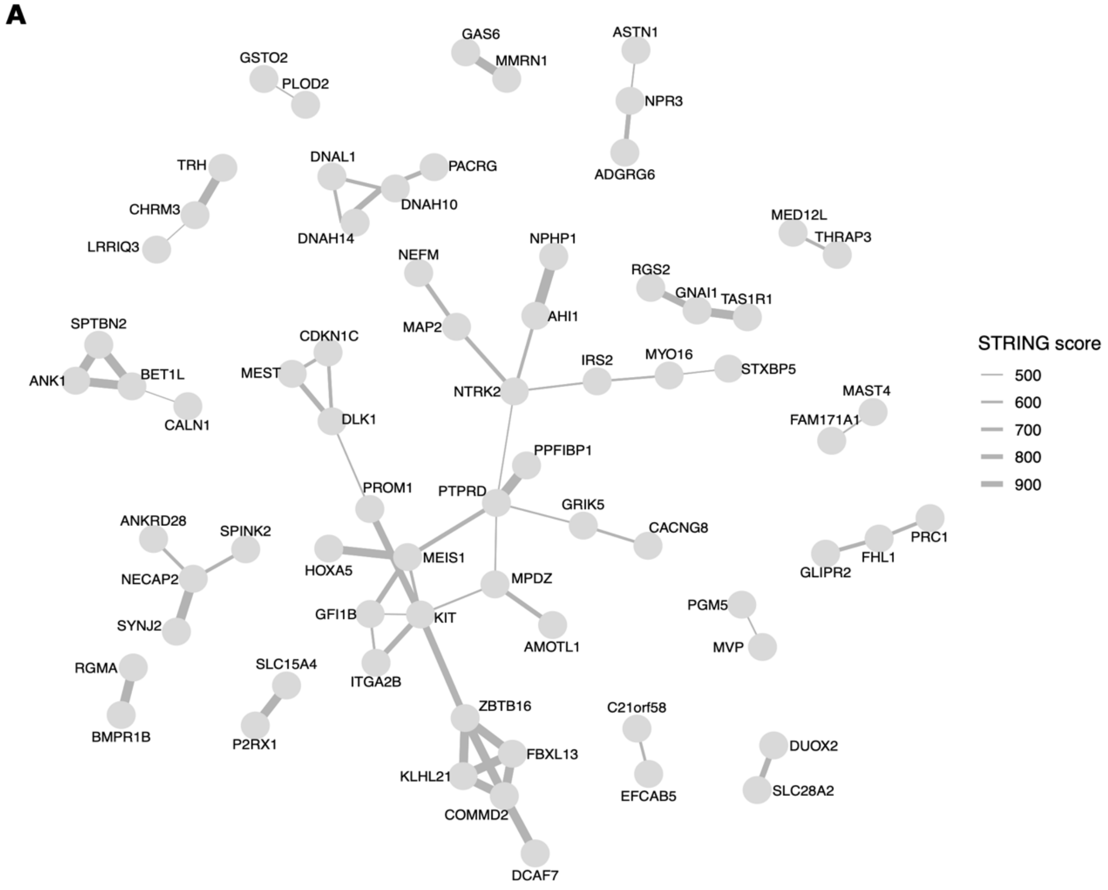

Figure S7. STRING network of media condition DEGs from monocyte transcriptional profile in MACDZ versus HC subjects. STRING network analysis of 138 Mav-independent monocyte DEGs which differentiate MACDZ vs HC subjects. Circles depict genes. Grey lines depict annotated connection between 2 genes in the STRING database with line thickness proportionate to the score.

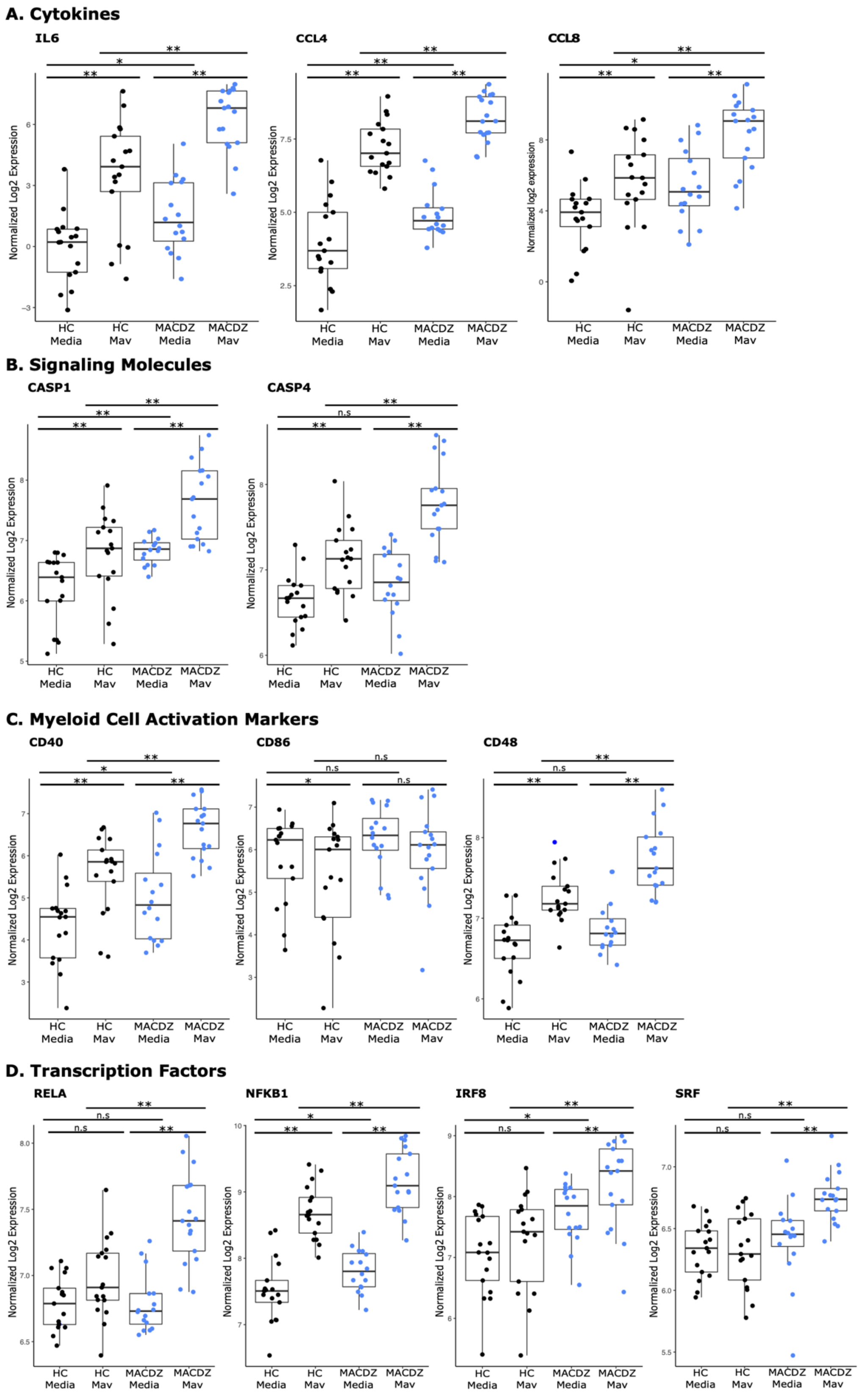

A. Cytokines
IL6
CCL4
CCL8
Normalized Log2 Expression
Normalized log2 expression
HC Media
HC Mav
MACDZ Media
MACDZ Mav
B. Signaling Molecules
CASP1
CASP4
n.s
C. Myeloid Cell Activation Markers
CD40
CD86
CD48
D. Transcription Factors
RELA
NFKB1
IRF8
SRF

Figure S8. DEG boxplots from monocyte transcriptional response to *Mycobacterium avium* infection in MACDZ versus HC subjects. Boxplots depicting voom normalized log2 mRNA expression values in MACDZ versus HC subjects with and without Mav infection. FDR values depict comparison of MACDZ vs HC media expression, MACDZ vs HC Mav expression, and Mav vs media for MACDZ or HC subjects (FDR≤0.1 shown by •, FDR≤0.05 shown by *, and FDR≤0.01 shown by **). Median and interquartile range depicted. Values were higher in the MACDZ subjects compared to HC for the media and/or Mav condition. Genes are expanded number and identical dataset as Fig. 4D-F.

Supplemental Tables

Tables 1-5 provided as excel files.

Table S1. List of peptide sequences included in the peptide library

Table S2. Differentially expressed genes identified in the RNAseq analysis of PBMCs

Table S3. Differentially expressed genes from linear model with interaction term comparing MACDZ versus HC monocyte transcriptional profiles with media and *M. avium* infection conditions. The table is split into three tabs for differentially expressed genes in MACDZ, Mav-dependent and interaction term groups, respectively.

Table S4. Gene set enrichment analysis with Hallmark mSigDB terms of MAC versus healthy control monocyte transcriptional profiles with media and *M. avium* infection conditions. The table is split into two tabs for pathways enriched in MACDZ vs. HC within *M. avium* infection and MACDZ vs. HC within media condition.

Table S5. Hypergeometric mean pathway enrichment analysis of 138 Mav-independent and 89 Mav-dependent DEGs of monocyte transcriptional profiles of MACDZ versus HC subjects with media and *M. avium* infection conditions. The table has separate tables for Hallmark and KEGG gene sets both for Mav-independent and Mav-dependent DEGs.
